# Prevalence and sociodemographic, clinical, and genetic characteristics of Fabry disease in north-central Chile, 2013–2023

**DOI:** 10.1016/j.ymgmr.2026.101313

**Published:** 2026-04-28

**Authors:** Sergio Royo Martínez, Mauricio Castillo-Montes, Fernando Molt, Basthian Cortes Rivera, Muriel Ramírez-Santana

**Affiliations:** a Unidad de Abastecimiento, Hospital San Pablo de Coquimbo, Coquimbo 1780000, Chile; b Departamento de Salud Pública, Facultad de Medicina, Universidad Católica del Norte, Coquimbo 1780000, Chile; c Servicio de Neurología, Hospital San Pablo de Coquimbo, Coquimbo 1780000, Chile; d Departamento de Clínicas, Facultad de Medicina, Universidad Católica del Norte, Coquimbo 1780000, Chile; e Servicio de Neurología Ambulatoria, Hospital San Pablo de Coquimbo, Coquimbo 1780000, Chile

**Keywords:** Fabry disease, Genetic diseases, Inborn, Prevalence, Cross-sectional studies, Rare diseases

## Abstract

**Background:**

Fabry disease (FD) is a rare genetic disorder linked to X chromosome, causing progressive multisystem involvement. Epidemiological data on FD are limited, both in Latin America and Chile. This study aimed to estimate the prevalence of FD and describe the sociodemographic and clinical characteristics of affected individuals in a north-central region of Chile.

**Methods:**

A descriptive cross-sectional study was conducted on 69 patients with FD during the period 2013–2023. An anonymized questionnaire collected data on sociodemographic variables, enzyme replacement therapy (ERT), chronic medications, clinical manifestations, and genetic variants. Descriptive statistics and correlation tests were used. Prevalence was estimated using official population data.

**Results:**

The regional prevalence rate was 8.44 per 100,000 inhabitants (95% CI: 6.44–10.43), with a particularly high rate in the commune of Coquimbo (22.45 per 100,000 inhabitants). The majority were women (58.0%), with a mean age of 35.8 years. 94% had the classical form of FD, and 98.6% carried the c.776C > G (p.P259R) variant. ERT was initiated in 2016 for 58% of patients, and 55.1% received agalsidase alfa. Peripheral nervous system involvement was present in 71.0%, and kidney involvement in 39.1%. The older the patient, the more systems are affected. 46.4% used complementary drugs, mostly analgesics, antihypertensives and antineuropathics, whose consumption increases with age.

**Conclusions:**

The Coquimbo Region has the highest prevalence of FD in Chile. Age at treatment initiation influences system involvement and chronic medication use. The results highlight the need for additional genetic, epidemiological, and clinical studies in the region.

## 1 Introduction

Fabry disease (FD), also known as Anderson-Fabry disease, is a lysosomal disease caused by variants in the GLA gene on the X chromosome. These variants cause a deficiency or absence of the lysosomal enzyme alpha-galactosidase A, leading to the progressive accumulation of globotriaosylceramide (Gb3 or GL-3) and other glycosphingolipids in the lysosomes. It is a rare systemic disease whose pathological process begins at an early stage, even during fetal development. However, most patients remain asymptomatic during the first years of life. The clinical manifestations of FD are broad and heterogeneous, and many of them can present as common symptoms.

Among the most common symptoms are skin lesions, particularly angiokeratomas, which are present in many cases. Cardiac involvement is observed in approximately 40 to 60% of patients, with cardiac muscle hypertrophy being one characteristic manifestation. Kidney disease is also common, with microalbuminuria, proteinuria and progressive deterioration of kidney function. From a neurological point of view, acroparesthesia as a manifestation of small fibre neuropathy is frequent. Ophthalmologically, whorled cornea, vascular tortuosity, and cataracts may be observed. In addition, neuro-otological manifestations have been described, such as progressive or sudden hearing loss. There is also involvement of the brainstem, the central nervous system, or the peripheral nervous system. Gastrointestinal disorders affect between 50 and 60% of patients, and bone and pulmonary complications may also occur [1], [2], [3].

FD occurs in two main forms: the classic and the non-classic form. The classic form, which has an early onset, is usually more severe and is caused by a complete or almost complete deficiency of the enzyme alpha-galactosidase A (activity <3%). It generally begins in childhood or adolescence and predominantly affects men, although it can also manifest in female carriers. On the other hand, the non-classical form (or late variant) has a later onset, usually presents a milder phenotype, and has variable clinical progression. In these cases, residual enzyme activity is higher (usually <30% in men). Patients may predominantly develop cardiac manifestations (such as cardiomyopathy), renal failure, or cerebrovascular accidents [3].

The diagnosis of FD is based on clinical signs and symptoms, which can be nonspecific and overlap with other pathologies, making it challenging to confirm on clinical grounds alone. For this reason, biochemical diagnosis includes measuring alpha-galactosidase A enzymatic activity in plasma or leukocytes, and genetic analysis to identify variants in the GLA gene. In addition, quantification of Lyso-Gb3 (globotriaosylsphingosine) in plasma, dried blood spots, and urine is used as a biomarker of lipid accumulation, useful for both diagnostic confirmation and monitoring treatment response [1].

Specific treatment of FD is based primarily on permanent enzyme replacement therapy (ERT) with agalsidase alfa or beta. Clinical evidence shows that the best results (including the prevention of irreversible damage) are achieved when ERT is initiated promptly. In addition, therapy with pharmacological chaperones, specifically oral migalastat, has been shown to be an effective and safe alternative in patients who meet the established therapeutic criteria. More recently, new enzyme replacement therapies such as pegunigalsidase alfa (a PEGylated recombinant α-galactosidase A with a prolonged half-life and reduced immunogenicity) have demonstrated favourable safety and efficacy profiles in clinical trials and have been approved in Europe and the United States, although they are not yet available in all regions, including Chile [4], [5]. On the other hand, many patients require supportive treatments or adjuvant therapies, which must be individualized according to clinical symptoms and the degree of involvement of the different organs and systems affected [6], [7].

Various studies worldwide have reported variations in prevalence, which depend on factors such as the methodology used, the population analyzed, and the type of diagnosis used. For example, in 2019, the prevalence was estimated to be 1 in 117,000 live births in Australia [8]. However, more recent research incorporating neonatal screening, genetic sequencing techniques, and biomarkers, in addition to measuring enzyme activity, has revealed higher prevalence estimates. In this regard, analysis of databases of pathogenic variants of the GLA gene in men and women has shown a population frequency of 1 in 3225 in the studied population, with a higher prevalence in women [9]. Recent multicenter studies have also reported a predominance of female patients, likely reflecting improved detection of heterozygous individuals, who were historically underdiagnosed [10], [11]. These findings suggest an upward trend in the prevalence of FD and reinforce the hypothesis that the disease continues to be underdiagnosed.

Knowing the prevalence and distribution of FD is essential to promote early detection, optimize clinical care, and properly guide public health policies. In this context, the Chilean Ministry of Health has, since 2016, established a protocol that provides specific guidelines for the entire network of healthcare providers treating patients with FD. This document standardizes the diagnostic procedure, inclusion criteria, clinical management, and administration of ERT [12].

In Chile, the characterization and prevalence of FD are still not precisely known. The only available figures come from the National Health Fund (FONASA) database, which records 139 beneficiaries with financial coverage for FD [13]. It is noteworthy that almost half of these patients are concentrated in the north-central part of the country, especially in the Coquimbo Region, where they are treated by a specialized multidisciplinary team at the San Pablo de Coquimbo Hospital. Consequently, the present study aims to estimate the prevalence of Fabry disease in a north-central region of Chile and to describe the sociodemographic, clinical, and genetic characteristics of the population studied.

## 2 Methods

A descriptive cross-sectional study was conducted to determine the prevalence and characterization of FD in the Coquimbo Region between 2013 and 2023. The sample included 69 patients with a confirmed diagnosis of FD, who received treatment and clinical follow-up at the San Pablo Hospital in Coquimbo, the regional referral center for this pathology, in accordance with the guidelines established by the Chilean Ministry of Health [12].

### 2.1 Context: Geographical, sociodemographic, and health system description of the Coquimbo Region

**Fig. 1:**
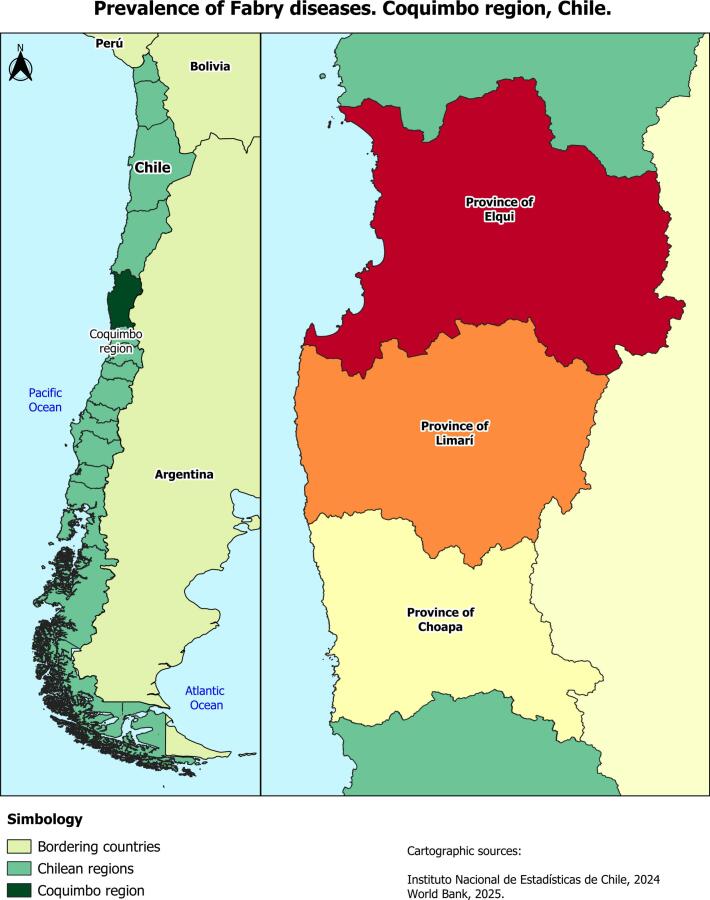
Map of Chile and neighboring countries highlighting the Coquimbo Region and its three provinces. Note: The colors distinguish Elqui, Limarí, and Choapa provinces; Elqui, shown in red, has the highest Fabry disease prevalence (11.8 per 100,000 inhabitants), Limarí shows an intermediate prevalence (2.75 per 100,000 inhabitants), and Choapa has no registered cases.

The Coquimbo Region is in north-central Chile, between 29°02′ and 32°16′ south latitude and from 69°49′ west longitude to the Pacific Ocean (Fig. 1, left side). Its capital is the city of La Serena, and it is subdivided into three provinces: Elqui (6 municipalities), Limarí (5 municipalities), and Choapa (4 municipalities) (Fig. 1). According to data from the 2024 Census [14], the regional population is 832,864, distributed mainly in the province of Elqui (562,457), followed by Limarí (178,057) and Choapa (92,350). 81.2% of the population resides in urban areas, while 18.8% live in rural areas [15]. In terms of social indicators, 7.9% of the regional population lived in poverty in 2022, a figure higher than the national average (6.5%). The employment rate was 64% for men and 40.9% for women [16].

Chile's healthcare system is mixed, consisting of a public insurer (FONASA), which covers approximately 77% of the national population [17], private insurers grouped into the Health Insurance Institutions (ISAPRE), and the Armed Forces' healthcare system. In 2015, Law No. 20,850 was enacted, establishing a financial protection system for high-cost diagnoses and treatments, applicable to beneficiaries of all social security schemes and financed by FONASA [18]. FD is one of the conditions covered by this law. The public health care network of the Coquimbo Region is organized by levels of complexity. It includes three high-complexity hospitals in the main urban centers and other medium- and low-complexity services across the region. Primary care facilities are widely distributed, with numerous centers and emergency units providing local access to care [19].

### 2.2 Health care of patients with FD: Suspicion, diagnosis, treatment, and follow-up

In the Coquimbo Region, the diagnosis and treatment of FD is centralized exclusively at the San Pablo Hospital in Coquimbo, the only facility in the region that has a neurology department and the Multidisciplinary Team for the Study and Treatment of Fabry Disease (EMETEF).

Patients with suspected or confirmed FD can access the Ministry of Health's public financial protection system for high-cost diagnoses and treatments, regardless of whether they are affiliated with the public (FONASA) or private health system, with access subject to evaluation and confirmation by an accredited committee of experts. There are two main ways to enter this system. The first is the diagnostic suspicion modality, aimed at patients with a well-founded suspicion of FD, usually formulated by medical specialists such as neurologists, internists, geneticists, or pediatricians. In men, the diagnosis is confirmed by measuring alpha-galactosidase A activity in leukocytes, supplemented by molecular genetic analysis. In women, due to phenotypic variability and the possibility of normal enzyme activity, molecular genetic testing is required for diagnostic confirmation. The second form of admission to the program is the therapeutic modality, which is aimed at patients with a previously confirmed diagnosis who require second-line or more complex treatment. Standard treatment consists of ERT with agalsidase alfa or agalsidase beta, which must be administered by authorized providers and is covered by Law No. 20,850 [12].

### 2.3 Participants, instruments, ethical aspects, data collection and analysis

The study included all 69 people diagnosed with FD in the Coquimbo Region between 2013 and 2023 who received treatment at the San Pablo Hospital in Coquimbo. For data collection, a questionnaire was designed using the Google Forms platform (Google LLC, Mountain View, CA, USA), without fields that would allow patient identification, to ensure confidentiality. The instrument was designed to be completed by the treating physician or a member of EMETEF and included instructions for its application and data recording. According to information provided by the attending physicians at the EMETEF, the patients are related. However, there is no record of the degree of family ties or specific relatedness.

The study protocol was approved by the Scientific Ethics Committee of the Faculty of Medicine of the Universidad Católica del Norte, under Resolution No. 56/2023. Institutional authorization was also obtained from the San Pablo Hospital in Coquimbo, the healthcare center where all patients with FD in the region are treated and whose clinical records are kept.

The questionnaire was completed by the healthcare team at the EMETEF using information contained in clinical records. The variables collected included: sex, age, municipality of residence, type of health insurance, year of initiation of therapy, type of enzyme replacement therapy, use of chronic medications, type of clinical manifestation, and type of genetic variant. The analysis of clinical manifestations focused on the main organs or systems affected: the peripheral nervous system (PNS), the renal system (RF), the heart (HT), and the central nervous system (CNS). Patients were classified by variant type, specifically by the presence or absence of the classic variant of the disease.

The prevalence of FD was estimated based on the 69 confirmed cases during the study period, without excluding deceased patients. The denominator used was the average regional population projection for the years 2013 to 2023, based on data from the National Institute of Statistics (INE) [20], and the 95% confidence interval was calculated.

For statistical analysis, contingency tables and the chi-square test (*p* < 0.05) were used to evaluate associations between sex and condition type, as well as between sex and chronic medication use. The comparison between independent groups -such as age, presence of specific clinical manifestations, and use of chronic medications- was performed using the Student's *t*-test, the nonparametric Mann-Whitney *U* test in cases where the assumption of homogeneity of variances was not met (*p* < 0.05). In addition, a nonparametric correlation (Spearman's Rho) was performed between the number of affected systems and the number of chronic medications consumed with age. Data processing and analysis were performed using JASP software version 0.18.3.0 and IBM SPSS Statistics version 26.

## 3 Results

The demographic and clinical characteristics of the 69 patients are detailed in Table 1. Most of the patients included in the study had the classic form of FD and carried the c.776C > G (p.P259R) variant (98.6%). Fifty-eight per cent were women, with a female-to-male ratio of 1.4:1. Participants' ages at therapy initiation ranged from 6 to 73 years, with a mean of 35.8 ± 18.67 years. 86% of patients were over 14 years of age. In terms of geographical distribution, the majority resided in the province of Elqui (92.8%), specifically in the municipality of Coquimbo (81.2%). See Fig. 1, right side. Regarding health insurance type, 97% were affiliated with the public system, and only 3% had private insurance.

**Table 1:** Characterization of patients with Fabry disease, Coquimbo Region, Chile, 2013–2023.

Variable	Category	Frequency	Percentage
Sex	Men	29	42.0%
	Women	40	58.0%
Age range of therapy initiation	0 to 14	12	17.4%
	15 to 29	17	24.6%
	30 to 44	19	27.5%
	45 to 59	11	15.9%
	60 to 74	10	14.5%
	75 and over	0	0.0%
Death rate		6	8.69%
Type of variant	c.776C > G (p.P259R)	68	98.55%
	c.243G > A (p.W81X)	1	1.45%
Province	Elqui	64	92.8%
	Limarí	5	7.2%
	Choapa	0	0,00%
Type of insurance	Public (FONASA)	67	97.1%
	Private	2	2.9%
Enzyme replacement therapy	Agalsidase alfa	38	55.1%
	Agalsidase beta	24	34.8%
	No TRE	7	10.1%
Type of clinic involvement	Peripheral Nervous System (PNS)	49	71.00%
	Renal System (RF)	27	39.1%
	Heart (HT)	26	37.7%
	Central Nervous System (CNS)	10	14.5%
Type of medication for chronic use	Analgesics	33	47.8%
	Antihypertensives	29	42.0%
	Antineuropathics	19	27.5%
	Hypolipidemic agents	16	23.2%
	Antiplatelet agents	14	20.3%
	Antiarrhythmics	7	10.1%
	Anticoagulants	4	5.8%
	Antivertiginants	2	2.9%

About the clinical manifestations and system involvement characteristic of FD, 71.0% of patients had PNS involvement, followed by RF involvement in 39.1%. No statistically significant differences were observed between sex and type of system involvement.

Regarding ERT, 58.0% of patients began treatment in 2016, coinciding with the entry into force of Law No. 20,850. However, by 2023, 10.1% had not yet begun ERT. Agalsidase alfa was used in 55.1% of cases, and among patients who received ERT, 61.3% were treated with agalsidase alfa and 38.7% with agalsidase beta. In addition to enzyme treatment, 46.4% of patients used complementary medications due to the type of involvement and the presence of chronic comorbidities, and in this group, women accounted for 69.5%. The most used drugs were analgesics (47.8%), antihypertensives (42.0%), and anti-neuropathic (27.5%).

Of the 69 patients diagnosed, 6 died during the study period, resulting in a fatality rate of 8.7%.

The prevalence rates of FD in the Coquimbo Region are presented in Table 2. The overall prevalence was 8.44 per 100,000 inhabitants (95% CI: 6.44–10.43), with a higher rate in women than in men. Among the three provinces in the region, Elqui had the highest prevalence, particularly in the commune of Coquimbo, with a rate of 22.45 per 100,000 inhabitants (95% CI: 16.57–28.33), the highest in the entire territory analyzed. In terms of age-group distribution, the highest prevalence rates were observed in the 30–44 and 60–74 age ranges, with similar values between the two.

**Table 2:** Prevalence of Fabry disease in the Coquimbo Region, Chile, 2013–2023.

Variable	Category	Population	Number of cases	Rate per 100,000 inhabitants	C.I. 95%	
					Lower	Upper
Sex	Men	400,573	29	7.23	4.6	9.87
	Women	416,895	40	9.59	6.62	12.56
Age range of therapy initiation	0 to 14	169,436	12	7.08	3.07	11.08
	15 to 29	179,244	17	9.48	4.97	13.99
	30 to 44	173,370	19	10.95	6.03	15.88
	45 to 59	152,399	11	7.21	2.95	11.48
	60 to 74	99,182	10	10.08	3.83	16.33
	75 and over	43,837	0			
**Elqui Province**		**542,112**	**64**	**11.8**	**8.91**	**14.69**
Commune	Coquimbo	249,437	56	22.45	16.57	28.33
	La Serena	242,699	4	1.64	0.03	3.26
	Vicuña	29,261	1	3.41	n.c.	n.c.
	Andacollo	11,698	1	8.54	n.c.	n.c.
	Paihuano	4631	2	43.18	n.c.	n.c.
	La Higuera	4386	0	0	0	0
**Limarí Province**		**181,813**	**5**	**2.75**	**0.33**	**5.16**
Commune	Ovalle	119,409	4	3.34	0.06	6.63
	Monte Patria	32,266	0	0	0	0
	Combarbalá	13,807	0	0	0	0
	Punitaqui	11,961	1	8.36	n.c.	n.c.
	Río Hurtado	4370	0	0	0	0
Choapa Province		93,543	0	0	0	0
**Total Coquimbo Region**		**817,468**	**69**	**8.44**	**6.44**	**10.43**

n.c. not calculable.

**Fig. 2:**
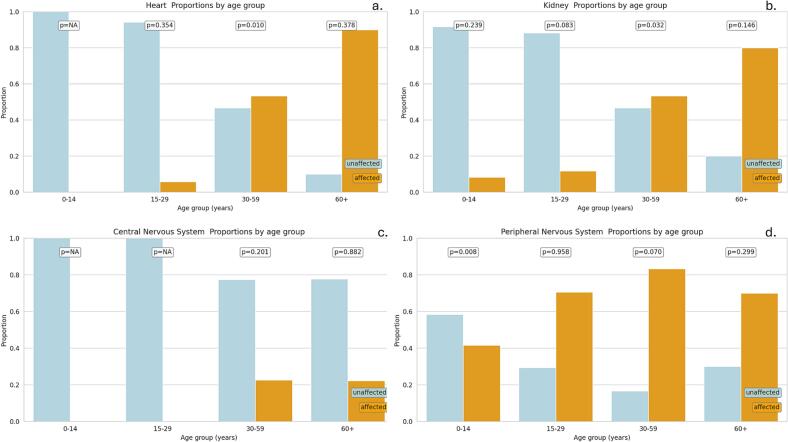
Proportion of clinical involvement by age group in patients with Fabry disease, Coquimbo Region, Chile, 2013–2023. Note: Affected: presence of clinical manifestations; Unaffected: absence of clinical manifestations. Panels show the proportion of affected and unaffected patients across age groups (0–14, 15–29, 30–59, and ≥ 60 years) for (a) heart involvement, (b) kidney involvement, (c) central nervous system involvement, and (d) peripheral nervous system involvement. *P*-values correspond to comparisons between affected and unaffected groups within each category.

**Fig. 3:**
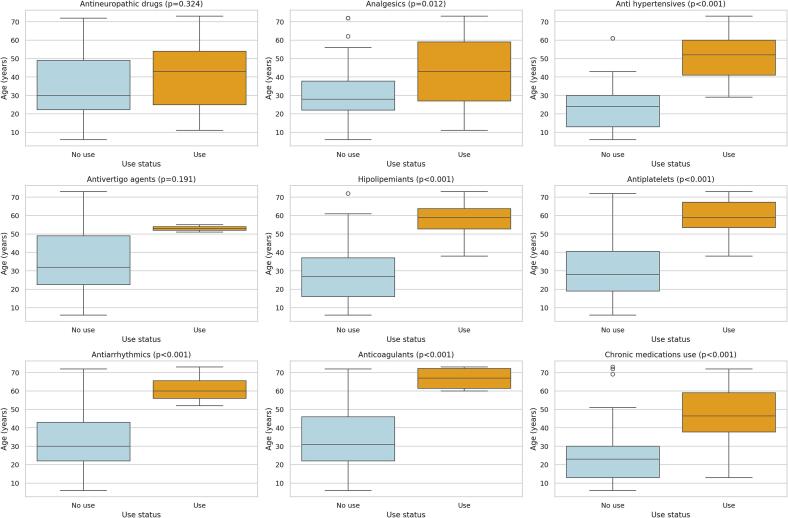
Age at the start of enzyme replacement therapy according to chronic use of medications for symptom management in patients with Fabry disease, ordered by increasing mean age in the use group, Coquimbo Region, Chile, 2013–2023. Note: Panels display boxplots comparing age at therapy initiation between patients who did and did not use each medication type (Mann-Whitney U test). Medications are ordered according to the mean age of patients in the use group, from lowest to highest, to reflect the sequence of therapeutic interventions across disease progression. Most medications were associated with older age at treatment initiation, except anti-neuropathic drugs (*p* = 0.324) and antivertigo agents (*p* = 0.191), for which no significant differences were observed.

Fig. 2, Fig. 3 present comparisons of ages at ERT initiation by the presence or absence of clinical manifestations and of chronic medication use, respectively. It was observed that patients with FD who presented some type of clinical condition had a significantly higher average age of therapy initiation than those without clinical manifestations. When analyzing the different types of conditions, it was found that a higher proportion of those with peripheral nervous system (PNS) involvement began treatment at a younger age, in contrast to those with central nervous system (CNS) involvement, who began treatment after the age of 30 (Fig. 2). These differences were statistically significant (see Supplementary Table 1). Likewise, differences in age at therapy initiation were observed for most chronically used medications for symptom relief, except for antineuropathic and antivertiginous drugs. In general, patients who took these medications were older at the start of treatment (Fig. 3).

Table 3 shows the correlation indices between the number of systems affected and the number of chronic medications, and between the age of treatment onset and the number of systems affected. It can be observed that age has a significant influence on both the number of systems affected and the number of chronic medications patients consume.

**Table 3:** Correlation between affected systems and chronic medications at the beginning of treatment, Coquimbo Region, Chile, 2013–2023.

Variable	Spearman's Rho	*p* value
Number of systems affected - age	0.68	<0.001
Number of drugs of chronic use - age	0.747	<0.001

## 4 Discussion

Although FD is classified as a rare or low-frequency pathology, its occurrence in the Coquimbo Region is of particular interest, as evidenced by this study's findings.

The prevalence observed in this study was 8.44 per 100,000 inhabitants in the Coquimbo Region, an elevated frequency, given that almost half of the cases registered in the country are concentrated there. This rate far exceeds those reported in various international studies. Reported prevalence of FD varies between 0.015 and 0.85 per 100,000 inhabitants [1], which may underestimate the true magnitude of the disease due to variability in the life expectancy of patients with FD and technical limitations in diagnosis [9]. Additionally, neonatal screening studies have revealed significantly higher figures. Prevalence at birth of 1 in 3225 has been estimated in Australia (equivalent to 31/100,000 inhabitants) [21], similar to the 1 in 3000 reported in USA [22], while in Japan, the neonatal prevalence was valued in 1.25/100,000 [23] and in Spain the prevalence in newborn was estimated in 13/100,000 [24]. In general, studies conclude that FD is globally underestimated, especially among women [21], [24].

Regarding sex, this study found a higher prevalence of FD in women than in men. While earlier studies, including those based on chronic kidney disease populations and neonatal screening programs, reported a higher frequency of FD in males than in females [24], [25], [26], more recent multicenter and cohort studies have shown an increasing proportion of affected women, likely reflecting improved detection of heterozygous individuals who were historically underdiagnosed [10], [11].

These differences can be explained by the disease's genetic basis, which is linked to the X chromosome. In men, who have a single X chromosome, clinical manifestations tend to appear earlier, with greater severity and accelerated progression. In women, however, the clinical presentation is more heterogeneous, with frequently subtle symptoms, later onset, greater survival, and variability in severity, attributable to random inactivation of the X chromosome. As a result, women tend to have higher enzyme levels and lower Lyso-Gb3 concentrations than men, making early diagnosis difficult [27], [28], [29]. The higher proportion of women observed in this study population may be explained by the identification of asymptomatic or less clinically evident female carriers through family screening of index cases, a strategy known to substantially increase case detection in FD. This pattern has been previously described, as identifying an index case can lead to the diagnosis of multiple affected relatives within the same family, thereby increasing case detection through cascade screening [30].

Over half of patients initiate treatment in their youth or early adulthood, and the average age is slightly lower in men than in women, which is consistent with reports from several previous publications [31], [32], [33].

The high prevalence of FD in the province of Elqui accounts for approximately 43% of all cases recorded nationwide, with most patients concentrated in the municipality of Coquimbo. It has been documented that the prevalence of FD can vary significantly across populations and geographical regions, depending on specific variants and predominant genetic patterns [34], [35]. In this study, over 98% of patients carry the c.776C > G (p.P259R) variant, suggesting a possible founder effect. Consequently, it is pertinent to investigate the origin and age of this variant through advanced genotyping studies and identification of shared haplotypes. These analyses would make it possible to determine the degree of consanguinity among cases reported in the province, as well as to evaluate the potential influence of minority ethnic components that may modulate the genomic architecture and clinical manifestations of the disease [36], [37].

Concerning ERT, no differences were observed in the frequency of use between agalsidase alfa and agalsidase beta. In Chile, treatment allocation is centrally regulated by the Ministry of Health (MINSAL), with patients assigned to either therapy in an alternating sequence rather than based on individual clinical prescription or physician preference. Therefore, treatment allocation does not necessarily reflect individual clinical decision-making. This centralized system may explain the similar distribution of therapies observed in our study. It should be noted that some patients diagnosed with FD did not receive enzyme replacement therapy, which may be explained by the absence of symptoms at the time of diagnosis, particularly among pediatric patients who did not yet meet the criteria established in the protocol [12]. However, clinical guidelines recommend initiating ERT early, even before irreversible organ damage occurs, to modify the disease's natural course, relieve pain, and improve quality of life. Several studies have shown that timely initiation of ERT can normalize plasma globotriaosylceramide (GL-3) levels, improve gastrointestinal symptoms, and reduce the incidence of renal and cardiac complications [38], [39]. This is consistent with the findings of the present study, which indicate that the older the age at diagnosis, the more systems are affected in patients. Six deaths were identified during the study period. Most were attributed to cardiovascular causes, while one was related to cancer. All deceased patients had received ERT and presented with multi-organ involvement. Detailed information on the circumstances of death was limited.

Given the multi-organ nature of FD, patients may require chronic use of one or more symptomatic medications, in addition to ERT. In this study, it was observed that almost half of the patients received additional pharmacological treatment, with analgesics being the most frequently used. This finding is probably related to the high prevalence of neuropathic pain, one of the characteristic and initial symptoms of FD. Likewise, greater use of complementary medications was observed with increasing patient age.

At the same time, patients with clinically affected systems were older at the start of their treatment. It has been reported that patients with FD may begin to show clinically relevant renal failure (RF) and heart manifestations (HT), such as left ventricular hypertrophy or hypertrophic cardiomyopathy, from the age of 30, with increasing progression and mortality in older age groups [40], [41], [42], [43], [44]. CNS involvement has also been linked to older ages, with symptoms ranging from headaches and dizziness to severe manifestations such as neuro-otological disorders or cerebrovascular events [45], [46]. On the contrary, patients with PNS involvement had a significantly lower average age (39.2 years) than those with RF, HT, or CNS involvement, whose average ages were higher. Similarly, people who used antineuropathic drugs also belonged to the younger age group, with no significant differences in age compared to those who did not receive such medication, and the effect size was small. This finding is consistent with the literature, which indicates that one of the first clinical manifestations of FD -often from childhood or adolescence- is acroparesthesia and Fabry neuropathic crises, characterized by acute neuropathic pain with a burning, stinging, or tingling sensation that can radiate to the distal extremities or other body regions [6], [47].

The existence of a specialized and multidisciplinary center for the care of FD at the Hospital San Pablo de Coquimbo is in line with the recommendations of experts in Latin America, who emphasize the need to strengthen the capacities of the health system for the timely diagnosis, comprehensive treatment and continuous follow-up of patients. Likewise, this type of initiative is consistent with policies that promote the financing of medication and comprehensive care for rare diseases [37].

### 4.1 Strengths and limitations

The main strength of the study is the minimal probability of patient loss, since all patients, both from public and private centers, are registered in the program and were included in the study. All diagnoses were validated by the EMETEF in accordance with the Ministry of Health Guidelines, and data were available for all cases, including the six deaths occurred during the period. The only potential source of loss may be people who have moved out of the region or are awaiting specialist evaluation in the final months of the study.

An important limitation of the study is that it was not possible to mislay the degree of blood relationship between patients, which limits the interpretation of the observed prevalence. A second limitation of the study is that, with the method used, it is not possible to calculate confidence intervals for prevalence rates in municipalities with small populations, where 1 or 2 cases can yield high rates and confidence intervals that extend into negative values. Finally, there is a limitation in recording patient age, as it reflects not the age at symptom onset but the age at which each patient enters the program and begins enzyme replacement therapy (ERT).

## 5 Conclusions

The prevalence of FD in north-central Chile is the highest recorded nationally, with a significant concentration of cases in the municipality of Coquimbo, among whom the majority express the same variant, suggesting consanguinity. Unlike what has been reported in the international literature, in this region, the disease predominantly affects women, which could be explained by the form of diagnosis. The age of treatment initiation significantly influences both the number of systems affected and the number of chronic medications consumed by patients, emphasizing the need for early diagnosis through screening and recommending genetic counseling for already identified patients.

The findings suggest conducting more in-depth research into the genomic representation of this population, with the aim of identifying potential shared origins and ethnic components that may influence the clinical expression and symptoms of FD. The region has a strategic advantage for this type of study, as it has a specialized, multidisciplinary center for FD at the San Pablo Hospital in Coquimbo, which serves as a regional reference for the diagnosis, treatment, and follow-up of this disease.

## Generative AI statement

During the preparation of this work, the authors used ChatGPT and Grammarly to assist with language editing and improvement of English expression. Julius AI was used exclusively for data visualization (box plot and bar charts design). After using these tools, the authors reviewed and edited the content as needed and take full responsibility for the published article.

## CRediT authorship contribution statement

**Sergio Royo Martínez:** Writing – original draft, Supervision, Resources, Project administration, Methodology, Investigation, Data curation, Conceptualization. **Mauricio Castillo-Montes:** Writing – review & editing, Writing – original draft, Visualization, Validation, Supervision, Methodology, Formal analysis, Conceptualization. **Fernando Molt:** Investigation, Data curation, Conceptualization. **Basthian Cortes Rivera:** Investigation, Data curation. **Muriel Ramírez-Santana:** Writing – review & editing, Visualization, Validation, Formal analysis.

## Ethics statement

This study was approved by the Scientific Ethics Committee of the Faculty of Medicine of the Universidad Católica del Norte (Res. CECFAMED-UCN REV N°56/2023).

## Funding

The authors did not receive funding from any organization for the presented research. The study was the thesis of the main author, to obtain the degree of Master's in Public Health.

## Declaration of competing interest

The authors declare that they have no conflicts of interest.

## Data Availability

The data supporting the findings of this study are not publicly available due to ethical and privacy restrictions related to patient confidentiality; aggregated data are publicly available.

## Appendix A Supplementary data

Supplementary material: Supplementary Table 1. Age at initiation of enzyme replacement therapy (ERT) according to clinical involvement and chronic medication use in patients with Fabry disease from the Coquimbo Region, Chile (2013–2023). This table complements the main results by providing detailed subgroup analyses.
