# Supplementary material for: Prevalence and sociodemographic, clinical, and genetic characteristics of Fabry disease in north-central Chile, 2013–2023
Source: Mol Genet Metab Rep. 2026 Apr 28;47:101313. doi: 10.1016/j.ymgmr.2026.101313 (PMC13140076; doi:10.1016/j.ymgmr.2026.101313)
Supplement: Supplementary file 1 — Supplementary material: Supplementary Table 1. Age at initiation of enzyme replacement therapy (ERT) according to clinical involvement and chronic medication use in patients with Fabry disease from the Coquimbo Region, Chile (2013–2023). This table complements the main results by providing detailed subgroup analyses. [file mmc1.docx]

**Supplementary Table 1:** Age at initiation of ERT according to clinical involvement and chronic medication use in patients with Fabry disease, Coquimbo Region, Chile, 2013-2023.

|  | **Characteristic** | **Group** | **n (%)** | **Mean ± SD** | **Median** | **p-value** | **Cohen´s d** |
| --- | --- | --- | --- | --- | --- | --- | --- |
| **Clinical involvement** | Peripheral Nervous System | Affected | 49 (71.0%) | 39.2 ± 17.6 | 40 | 0.015 | 0.285 |
|  |  | Unaffected | 20 (29.0%) | 27.2 ± 19.2 | 26 | - | - |
|  | Renal Function | Affected | 27(60.9%) | 49.7 ± 15.7 | 51 | <0.001 | 0.297 |
|  |  | Unaffected | 42(39.1%) | 26.7 ± 14.7 | 26 | - | - |
|  | Heart | Affected | 26(37.7%) | 52.8 ± 13.0 | 52.5 | <0.001 | 0.332 |
|  |  | Unaffected | 43(62.3%) | 25.4 ± 13.5 | 26 | - | - |
|  | Central Nervous System | Affected | 10(14.5%) | 52.8 ± 12.0 | 52 | 0.001 | 0.358 |
|  |  | Unaffected | 59(85.5%) | 32.8 ± 18.2 | 30 | - | - |
| **Medication use** | Antineuropathic drugs | Use | 19 (27.5%) | 39.4 ± 19.0 | 43 | 0.324 | 0.271 |
|  |  | No use | 50 (72.5%) | 34.3 ± 18.7 | 30 | - | - |
|  | Analgesics | Use | 33 (47.8%) | 41.6 ± 18.9 | 43 | 0.012 | 0.252 |
|  |  | No use | 36 (52.2%) | 30.3 ± 17.2 | 28 | - | - |
|  | Antihypertensives | Use | 40 (42.0%) | 51.5 ± 12.9 | 52 | <0.001 | 0.323 |
|  |  | No use | 29 (58.0%) | 24.3 ± 13.2 | 24 | - | - |
|  | Antivertigo agents | Use | 2 (2.9%) | 53 ± 2.8 | 53 | 0.191^a^ | 0.395^a^ |
|  |  | No use | 67 (97.1%) | 35.2 ± 18.8 | 32 | - | - |
|  | Hipolipemiants | Use | 16 (23.2%) | 58.1 ± 10.0 | 59 | <0.001 | 0.348 |
|  |  | No use | 53 (76.8%) | 29 ± 15.2 | 27 | - | - |
|  | Antiplatelets | Use | 14 (20.3%) | 58.4 ± 10.5 | 59 | <0.001 | 0.35 |
|  |  | No use | 55 (79.7%) | 30 ± 15.8 | 28 | - | - |
|  | Antiarrhythmics | Use | 7 (10.1%) | 61.1 ± 7.7 | 60 | <0.001^a^ | 0.441 |
|  |  | No use | 62 (89.9%) | 32.9 ± 17.5 | 30 | - | - |
|  | Anticoagulants | Use | 4 (5.8%) | 66.7 ± 6.7 | 67 | <0.001 | 0.542 |
|  |  | No use | 65 (94.2%) | 33.8 ± 17.6 | 31 | - | - |

**Note:** Data are presented as mean ± standard deviation (SD) and median. Group comparisons were performed using the Mann-Whitney U test. When the assumption of homogeneity of variances was not met, Welch´s t-test was applied (^a^). A p-value < 0.05 was considered statistically significant.
